# Occurrence and relevance of *Gardnerella vaginalis* in urine cultures of kidney transplant recipients- a retrospective cohort analysis

**DOI:** 10.1186/s12866-026-05522-6

**Published:** 2026-09-01

**Authors:** Sabrina Klein, Silke Deininger, Iris Schröter, Dennis Nurjadi, Claudia Sommerer

**Affiliations:** 1 Department of Infectious Diseases, Heidelberg University, Medical Faculty Heidelberg, Medical Microbiology and Hygiene, Heidelberg, Germany; 2 Department of Infectious Diseases, Heidelberg University Hospital, Medical Microbiology and Hygiene, Im Neuenheimer Feld 324, Heidelberg, 69120 Germany; 3 University of Luebeck, Institute of Medical Microbiology, Lübeck, Germany; 4 University Hospital Schleswig-Holstein, Campus Luebeck, Institute of Medical Microbiology, Luebeck, Germany; 5 German Center for Infection Research (DZIF), Partner Site Hamburg-Lübeck-Borstel-Riems, Luebeck, Germany; 6 Department of Nephrology, Heidelberg University Hospital, Heidelberg, Germany; 7 German Center for Infection Research (DZIF), Partner Site, Heidelberg, Germany

**Keywords:** *Gardnerella vaginalis*, Kidney transplantation, Urinary tract infection, Urine culture

## Abstract

**Background:**

*Gardnerella vaginalis* is known to be associated with the development of bacterial vaginosis. In contrast, its cultivation from urine specimen in significant number is not generally regarded as causative for urinary tract infections. In a previous study, we detected *G. vaginalis* predominantly in urine cultures of renal transplant recipients and questioned whether this is of relevance. To address this, we conducted a retrospective cohort study over a period of two years on renal transplant recipients who presented at the outpatient clinic of the Department of Nephrology of Heidelberg University Hospital. Patient characteristics and laboratory parameters of renal transplant recipients with *G. vaginalis* in urine cultures were compared to those of renal transplant recipients presenting in the same outpatient clinic with urine specimen culturing other bacteria than *G. vaginalis*, using a multivariable logistic regression analysis.

**Results:**

During the study period, *G. vaginalis* was detected in 70 urine samples of 38 patients. The majority of patients was female (86.8%, *n* = 33/38), and the age was significantly lower than in the control group (median 41 years and 51 years; OR 0.94, 95% CI 0.92–0.98, *p* = 0.001). a significant number of culture-forming units (CFU, ≥ 10^5^ /ml) were detected in half of the samples containing *G. vaginalis*; a second microorganism was cultured in 64.3% (*n* = 45/70), while more than two species grew in 53.9% (*n* = 82/152) of the control group samples. Besides age, bacterial load of ≥ 10^5^ CFU/ml was significantly associated the presence of *G. vaginalis* in urine cultures (OR 2.71, 95% CI 1.12–6.56, *p* = 0.003). The medical record was lacking information on clinical signs and symptoms of urinary tract infections.

**Conclusion:**

As clinical symptoms were not documented, the occurrence of *G. vaginalis* in urine samples of kidney transplant recipients cannot be finally assessed*.* Further studies are needed to clarify its significance, particularly in immunocompromised hosts.

## Background

*G. vaginalis* is a Gram-positive rod that has initially been described as “*Hemophilus vaginalis”* causing “nonspecific vaginitis” by Gardner and Dukes 1959 [1]. Since then, its role in the development of bacterial vaginosis has been well studied and it is clear, that it can be found as part of the vaginal flora in symptomatic and asymptomatic individuals [2, 3]. It is known to be involved in the development of bacterial vaginitis and research has focused on this disease to elucidate pathogenetic mechanisms [3]. Apart from bacterial vaginitis, various reports on extragenital infections caused by *G. vaginalis* have been published, among them bloodstream infections [4–13], demonstrating its pathogenic potential. In most of the cases, the patients were immunocompromised by different aetiologies, or had an underlying disease involving the genital or urinary tract.

While the presence of *G. vaginalis* in urine cultures has been documented for decades [14–17], its clinical significance remains controversial, as its presence is often interpreted as contamination with vaginal flora rather than indicating an urinary tract infection [18, 19]. Of note, investigations on the urinary microbiome demonstrate that the bladder is rather colonized by a complex microbial community than being sterile, even in asymptomatic and healthy individuals [20–22]. Studies applying extended cultures and culture-independent methods also identified *Gardnerella* spp. in the urinary microbiome of asymptomatic and symptomatic individuals [20, 21, 23].

Different methods for the typing of *G. vaginalis* have been developed and applied over the years and allow the description of biotypes, clades and genomospecies [24–27]. Although several studies aimed to describe association of different types with specific clinical presentations, results vary between studies [26].

In a previous study, we demonstrated that processing urine samples with total laboratory automation led to significantly more urine cultures growing *G. vaginalis*. This effect was especially prominent in urine specimen of kidney transplant recipients [28]. Being immunocompromised, these patients are prone to infections in general; and urinary tract infections are found to be associated with acute kidney injury which may lead to graft loss [29, 30]. In addition, they may present with atypical or even without clinical signs [31], making interpretation of urine culture findings particularly challenging. Although current guidelines question the relevance of *G. vaginalis* also in these immunocompromised patients [18], data addressing its clinical relevance in kidney transplant recipients remain limited.

Therefore, we conducted a retrospective cohort analysis to determine the relevance of *G. vaginalis* in urine cultures of kidney transplant recipients and analyse possible associated factors.

## Methods

### Study population and setting

Renal transplant recipients presenting in the outpatient clinic of the Department of Nephrology at Heidelberg University Hospital between July 2016 and July 2018 were retrospectively screened for the presence of *G. vaginalis* in urine culture results. Inclusion criteria were: patients who had previously undergone a renal transplantation; who were cared for in the outpatient clinic of the Department of Nephrology; and had at least one positive urine culture result for *G. vaginalis* during the study period. Inclusion of repeated cultures was allowed. Patients who did not meet the criteria above were excluded from the study. Patients with incomplete documentation could be included in the study, if they met the inclusion criteria.

The study was designed as a pilot study as the investigated parameters have not been assessed in this setting before. Based on an earlier study, where 99 urine samples growing *G. vaginalis* in a 6-month period with 25% of samples retrieved form kidney transplant recipients [28], the study was designed to assess about 100 cases retrospectively in a 2-year period.

**Fig. 1:**
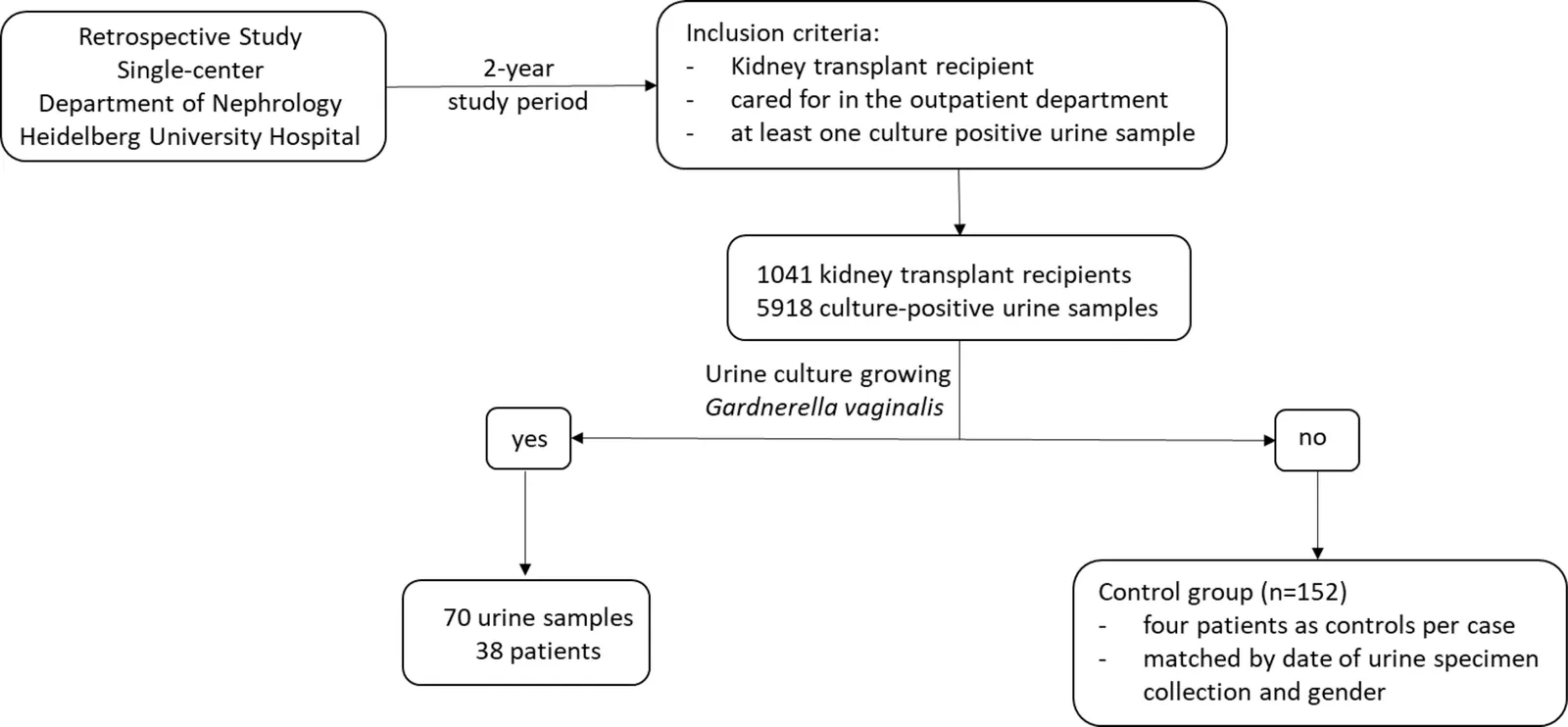
Study setting and inclusion criteria

Renal transplant recipients presenting in the same outpatient clinic without urine cultures culturing *G. vaginalis* were randomly assigned to serve as a control group. Randomization strategy was as follows: The list of patients of the outpatient clinic without presence of *G. vaginalis* was sorted by the day of urine specimen collection. For each patient with *G. vaginalis*, four gender-matched patients with a urine sample collected at the same day were randomly assigned to serve as control with the help of http://www.randomizer.org. Visualisation of inclusion criteria is provided in Fig. 1.

Of the selected patients, age, gender, time since transplantation and number of transplantations, medication, urine culture results, clinical signs of urinary tract infections and laboratory parameters (C-reactive protein (CrP), leucocyte count in urine and blood, glomerular filtration rate (GFR) and haemoglobin (Hb)) were analysed and compared between patients with *G. vaginalis* and the control group by a multivariable logistic regression including the first episode of a positive urine culture. Furthermore, the correlation of bacterial load as CFU/ml and leukocyte count in urine was assessed using Spearman`s rank correlation coefficient, including all samples. Clinical signs of urinary tract infections were defined according to current guidelines dysuria, urgency, frequency and/or suprapubic pain [32]. All urine samples included in the study were midstream urine and processed in the routine microbiological diagnostic as previously described [28]. In brief, urine samples were processed using BD Kiestra Total Laboratory Automation system with inoculation of 10 µl urine on Columbia agar with 5% sheep blood (BD Diagnostics, Heidelberg, Germany) and CPS agar (bioMérieux, Marcy l’Etiole, France) and incubated with 5 ± 1% CO_2_ for 24 h until imaging and reading. Isolates were identified by MALDI-TOF (Bruker Diagnostics, Billerica, MA) with a score ≥ 2 determining the bacterial species. Antimicrobial susceptibility testing on *G. vaginalis* isolates was not performed in this routine diagnostic setting.

For visualization and descriptive statistics, GraphPad Prism version 10.6.1 (SanDiego, California) and Stata (Stata Statistical Software, College Station, Texas) were used.

## Results

During the study period, 5918 urine samples of 1041 kidney transplant recipients were sent for microbiological analysis and *G. vaginalis* was cultured in 70 urine specimens (1%) of 38 patients (3%). Documentation of patient characteristics, immunosuppressive therapy, laboratory parameters as defined was complete for all patients assessed. Clinical symptoms of UTI were not documented in any of the cases.

### Patient characteristics

Among the patients with urine cultures growing *G. vaginalis*, 86.8% (*n* = 33/38) were female and the age was 41 years in median (range 23 to 65 years). In the control group, 84.2% were female (*n* = 128/152), with an age of 51 years in median (range 22–78 years). As gender was one criterium for randomization, the percentage of males and females was equal in both groups. The age in the groups differed significantly (Table 1, multivariate logistic regression, *p* = 0.001).

**Table 1:** Overview over patient characteristics, immunosuppression and laboratory parameters

**n**				**crude**			**adjusted**		
**Patient characteristcs**		***G. vaginalis***	**Control group**	**odds ratio (OR)**	**95% CI**	**p-value**	**odds ratio (OR)**	**95% CI**	***p*-value**
	Age (years)^a^	41 (33–52)	51 (39–61.5)	**0.96**	0.94–0.99	**0.003**	**0.94**	0.92–0.98	**0.001**
	female	*n* = 33/38 (86.8%)	*n* = 128/152 (84.2%)	1.23	0.43–3.49	0.7	0.83	0.24–2.94	0.78
	> 1 kidney transplantation	*n* = 3/38 (7.8%)	*n* = 10/152 (6.6%)	1.22	0.32–4.66	0.8	0.59	0.13–2.74	0.51
	days since transplantation^a^	2659.5 (1176–4312)	1949 (649–3631.5)	1.00	0.99–1.00	0.3	1.00	0.99–1.00	0.34
Immunosuppression									
	ciclosporin	*n* = 18/38 (47.4%)	*n* = 59/152 (38.8%)	1.42	0.69–2.90	0.3	4.27	0.19–91.5	0.35
	prednisolone	*n* = 34/38 (92.1%)	*n* = 134/152 (88.2%)	1.14	0.36–3.59	0.8	0.44	0.01–14.0	0.65
	mycophenocil acid	*n* = 32/38 (84.2%)	*n* = 127/152 (83.6%)	1.05	0.40–2.77	0.9	0.85	0.14–5.25	0.86
	tacrolimus	*n* = 16/38 (42.1%)	*n* = 71/152 (46.7%)	0.83	0.40–1.70	0.6	4.49	0.22–91.6	0.33
Laboratory parameter									
	bacterial load ≥ 10^5^ CFU/ml	*n* = 15/38 (39.5%)	*n* = 33/152 (21.7%)	**2.35**	1.10–5.00	**0.03**	**2.71**	1.12–6.56	**0.03**
	CrP (mg/l)^a^	2.0 (2.0–6.1)	2.0 (2.0–7.7)	1.00	0.99–1.00		0.99	0.99–1.00	0.66
	Leu/blood (/nl)^a^	8.2 (6.0–9.3)	7.3 (5.7–9.7)	1.00	0.99–1.01	0.51	0.99	0.99–1.01	0.88
	Leu/urine^a^	10 (4–22)	7 (0–20)	1.00	0.99–1.00	0.23	1.00	0.99–1.00	0.44
	GFR^a^	39 (26–53)	49 (36–66.5)	**0.98**	0.96–0.99	**0.02**	0.98	0.96–1.00	0.06
	Hb (g/dl)^a^	11.9 (10.1–12.9)	12.0 (11.0–13.1)	0.98	0.96–1.00	0.14	0.99	0.97–1.03	0.86

^a^median (interquartile range)

Of the patients with *G. vaginalis*, 92.1% (*n* = 35/38) had undergone a single transplantation and the remaining patients had a (*n* = 3/38) second transplantation. In the control group, 93.4% (*n* = 142/152) had the first transplantation, 4.6% (*n* = 7/152) a second transplantation and the remaining patients (*n* = 3/152) a third transplantation. Thus, the percentage of patients after the first transplantation was comparable in both groups.

### Immunosuppression

Most patients received standard triple immunosuppressive therapy consisting of a calcineurin inhibitor (ciclosporin or tacrolimus), prednisolone, and mycophenolic acid (*n* = 163/190, 85.7%). There was no significant association regarding the individual immunosuppressive agents (Table 1).

### Urine culture results and laboratory findings

The median time until the first urine sample was obtained that grew *G. vaginalis* was 2659.5 days (Interquartile range (IQR) 3136; 87 months; Table 1);) after the first transplantation. Of the 38 patients with a urine sample culturing *G. vaginalis*, seven patients (18.4%) had another urine culture with *G. vaginalis* and five (13.1%) three urine cultures. One patient had eight cultures with *G. vaginalis* over a period of four months and one patient nine urine cultures over a time period of eight months.

As the bacterial load in a urine sample expressed as colony forming units (CFU) per millilitre is one microbiological criterium to distinguish contaminating species from relevant pathogens, we compared the CFU/ml in the samples of both groups (Table 2). About half of the urine specimen with *G. vaginalis* showed a significant quantity with 10^5^ CFU/ml or more, which was significantly influencing the positivity of the sample (multivariable logistic regression restricted to the first sample in the *G. vaginalis* group, Table 1). In the control group, about two third of the samples showed a quantity of 10^4^ CFU/ml or less. The cultured species in the control group with the respective CFU/ml are displayed in Table 2.

**Table 2:** Colony forming units per millilitre and cultured microorganism from all urine specimen in the study

CFU/ml	*G. vaginalis* group		control group		microorganisms cultured in the control group
	n	%	n	%	
10^2^	1	1.4	38	25.0	Coagulase-negative Staphylococci (*n* = 8); Enterobacterales (*n* = 5); *Streptococcus* spp. (*n* = 6); *Enterococcus* spp. (*n* = 3); > 2 species (*n* = 16)
10^3^	14	20.0	43	28.3	*E. coli* (*n* = 4); *Enterococcus* spp. (*n* = 4); *K. pneumoniae* (*n* = 1), *C. freundii* (*n* = 1); *E. aerogenes* (*n* = 1); Coagulase-negative Stahylococci (*n* = 2); *Streptococcus* spp. (*n* = 3); > 2 species (*n* = 27)
10^4^	20	28.6	38	25.0	*E. faecalis* (*n* = 2); *Alloscardovia omnicolens* (*n* = 1); *C. koseri* (*n* = 1); *Lactobacillus* spp. (*n* = 2); Coagulase-negative Staphylococci (*n* = 1); > 2 species (*n* = 31)
10^5^	30	42.9	9	5.9	*E. faecalis* (*n* = 2); *Aerococcus urinae* (*n* = 1); *Lactobacillus jensenii* (*n* = 1); > 2 species (*n* = 5)
> 10^5^	5	7.1	24	15.8	*E. coli* (*n* = 12); *E. faecalis* (*n* = 4); *S. agalactiae* (*n* = 2); *P. mirabilis* (*n* = 1); *S.* *anginosus* (*n* = 1); *L. jensenii* (*n* = 1;) > 2 species (*n* = 3)

Cultures with > 2 species or 10^2^ CFU/ml were not differentiated further

**Fig. 2:**
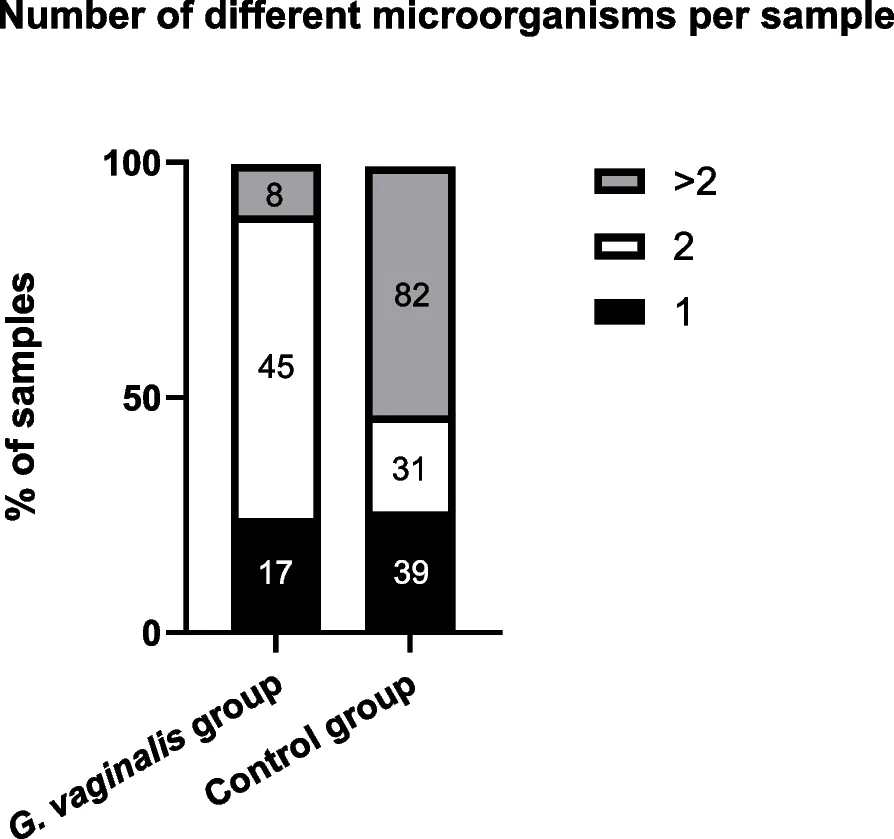
Number of different microorganisms per sample

Another criterium to assess whether urine samples are contaminated is the number of cultured microorganisms. For this analysis, all urine cultures culturing *G. vaginalis* were included, not only the first episode of each patient. The percentage of monocultures was similar in both groups with (24.3% of samples (*n* = 17/70) in the group with *G. vaginalis* and 25.7% of samples (*n* = 39/152) in the control group (Fig. 2). Figure 2 The numbers in the bars represent the number of samples. The total numbers of samples for *G. vaginalis* group was 70; and for the control group 152. All samples were included.

The majority of urine specimen with *G. vaginalis* cultured another microorganism (64.3%, *n* = 45/70), and in the control group this was about 20.4% (*n* = 31/152). Most of the samples in the control group (53.9%, *n* = 82/152) showed growth of more than 2 microorganisms, which was only the case in 11% (*n* = 8/70) of samples growing *G. vaginalis*.

In the control group, the most prominent pathogen in monocultures was *Escherichia coli* (*n* = 10/39), followed by *Enterococcus faecalis* (*n* = 6/39), several Enterobacterales (*Proteus mirabilis*, *Citrobacter freundii*, *Klebsiella aerogenes* and *Klebsiella pneumoniae*; *n* = 7). The remaining pure cultures were *Aerococcus urinae* (*n* = 1), *Enterococcus* spp. (*n* = 3), Coagulase-negative Staphylococci (CNS, *n* = 7), *Lactobacillus* spp. (*n* = 1) and α-hemolytic Streptococci (*n* = 4). In cultures growing 2 different species, Enterobacterales were grown in 10 samples, *Enterococcus* spp. in 7 samples, α-hemolytic Streptococci in 4 samples and *Lactobacillus* spp. in 4 samples. The remaining urine specimen grew Gram-positive Cocci including CNS.

In urine cultures growing *G. vaginalis* with another microorganism, the most prevalent pathogen was *Streptococcus agalactiae* (*n* = 10), followed by α-hemolytic Streptococci (*n* = 17), CNS (*n* = 6) and *Lactobacillus* spp. (*n* = 3).

**Fig. 3:**
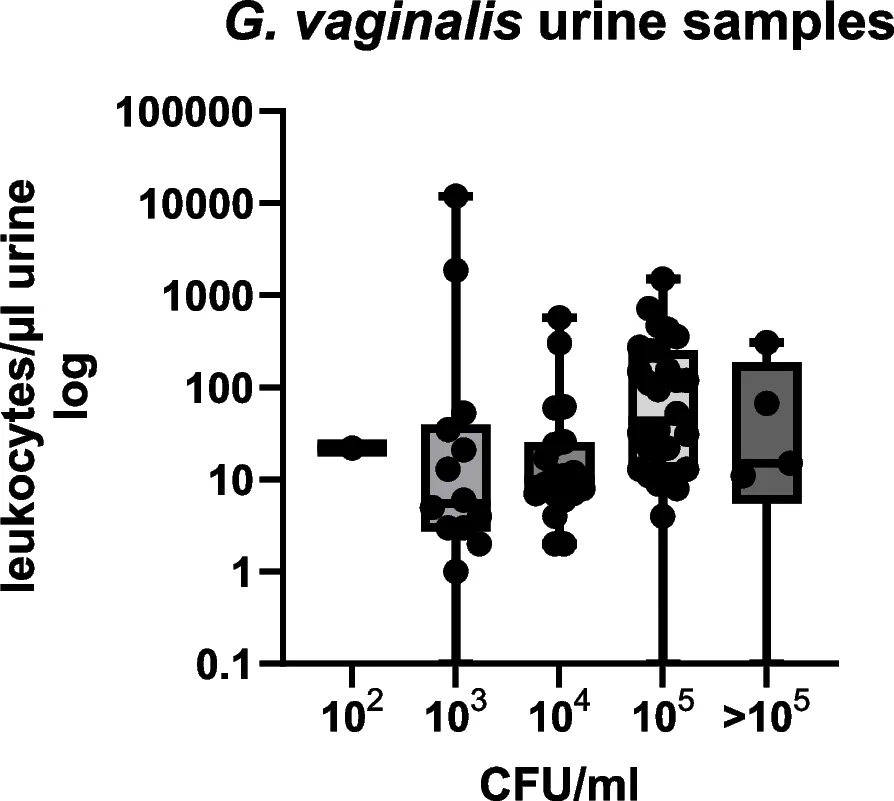
Leukocyte count in urine and colony forming units *of G. vaginalis*

Leukocyte count in urine and the corresponding CFU/ml in the respective sample are displayed in Fig. 3. Urine leukocyte counts correlated moderately with CFU/ml (Spearman r = 0.3232 [0.08843–0.5239], *p* = 0.006). There was no association with leukocyte count in urine, C-reactive protein (CrP), blood leukocyte count, glomerular filtration rate (GFR) or haemoglobin (Hb) in the adjusted multivariate regression, while there was a moderate association with the glomerular filtration rate in the crude analyses. (Table 1). Figure 3 All samples culturing *G. vaginalis* are displayed with a single dot representing a single sample. Spearman r = 0.3232 [0.08843–0.5239], *p* = 0.006.

## Discussion

In a previous work, we detected *G. vaginalis* in urine cultures predominantly of kidney transplant recipients [28] and questioned whether this is of relevance in this specific population. Following renal transplantation, patients are in general prone to infections due to the required immunosuppression. Especially UTIs may lead to ascending infections, cause transplant failure and lead to worse outcomes [29, 31], which may lower the threshold to consider atypical organisms such as *G. vaginalis* as clinically relevant. On the other hand, asymptomatic bacteriuria should not be treated in these patients according to current guidelines [32, 33].

In general, it is widely accepted that the origin of pathogens causing UTIs is usually the microflora of the urogenital tract, which involves the female vaginal flora [19]. Thus, it seems reasonable that facultative pathogenic organisms like *G. vaginalis* has the potential to cause UTIs, as well. Yet, current guidelines classify *G. vaginalis* as contaminant of urine samples [18].

Several factors help to distinguish between contamination by urethral flora and relevant pathogens in urine samples obtained through non-invasive procedures like midstream urine [34]. First, clinical symptoms including dysuria, frequency and urgency or suprapubic pain should be present [35–37]. Second, the presence of leucocytes in urine may be a sign of inflammation in the urinary tract, and nitrite can be detected as a product of bacterial metabolisms [34, 35]. Furthermore, the number of bacteria cultured, specified as CFU per millilitre, is a diagnostic criterium. Moreover, as UTIs are expected to be caused by one bacterial species, the presence of more than one microorganism may be a sign of contamination with urethral flora [34, 38]. However, none of these findings alone reliably distinguishes contamination from clinically relevant infection; particularly for organisms not classically considered uropathogens such as *G. vaginalis*.

Our study population was predominantly female and had a lower age compared to the control group, which is consistent with the presumed origin from the premenopausal vaginal flora. Approximately half of the specimens showed ≥ 10^5^ CFU/ml *G. vaginalis*, a bacterial load that would be unusual for incidental contamination alone; and high bacterial load was associated with a specimen culturing *G. vaginalis* in our analysis. However, high bacterial load may rather be the prerequisite for its cultural detection than implying causality. Furthermore, it has been demonstrated that high numbers of bacteria can be recovered from urine specimen of asymptomatic individuals with the use of extended cultures [20, 39]. Moreover, the presence of bacteria in urine samples might be reflecting the urinary microbiome, as this has been demonstrated also with the use of culturomics and metagenomic approaches [21]. These findings challenge the assumption of a sterile bladder in healthy individuals that has been guiding microbiological diagnostics and interpretation of culture results of UTI since decades [22]. Anyhow, the majority of urine cultures growing *G. vaginalis* cultured more than one microorganism, and those found concomitantly in urine cultures are mostly belonging to the vaginal and/or urethral flora, which may point to a possible contamination of the samples. Alternatively, this may be reflecting the urinary microbiota of the bladder, which may also be present in kidney transplant recipients. Indeed, the bacterial species that were cultured in our population were also detected in other studies [20, 39, 40]. On the other hand, the proportion of urine samples with more than one and more than two different microorganisms was even higher in the control group, weakening the argument that mixed growth alone indicates contamination. Therefore, polymicrobial growth should not automatically lead to dismissal of *G. vaginalis* as clinically irrelevant, particularly in immunocompromised patients. It should also be considered, that Gram-positive bacteria may not only be uropathogenic themselves, but interact with and support other uropathogens [19].

Recent studies highlight the limitations of conventional, culture-based detection of potential uropathogens. Applying culturomics and culture independent methods like metagenomic sequencing, the detection of fastidious, slow-growing or anaerobic bacteria in urinary tract samples [21, 39, 41]. This also included *Gardnerella* spp. that can be detected in 2 −21% of samples retrieved from patients with symptoms of UTI [21, 42]. Due to the retrospective observational nature of our study, the urine samples were not preserved and not available for further analyses, for example with metagenomic sequencing. This therefor limits potential complementary information that could be obtained with another method. In addition, bacterial isolates were not available to assess antimicrobial susceptibility or potential associations with specific genotypes.

A relevant aspect for the diagnosis of UTI is the presence of clinical symptoms [36, 37]. In the absence of clinical symptoms, urine specimen would usually not be assessed; but if so, patients would be judged to have an asymptomatic bacteriuria [32, 34, 35]. In our study, absence or presence of typical symptoms was unfortunately not documented, which may suggest their absence, but cannot be used to judge on clinical relevance of the findings.

Our study has several limitations. Due to the retrospective character and the limited number of patients in our centre, conclusions on clinical outcomes associated with *G. vaginalis* cannot be drawn. This would need to be assessed in a multicentre prospective study, possibly involving an intervention. In addition, regular screening urine cultures in transplant recipients may introduce selection bias compared to routine diagnostic approaches in the general population [32]. To address this flaw given by the setting, we chose a control group in the cohort of kidney transplant recipients and compared characteristics of those with *G. vaginalis* in urine culture to those without. This allowed an unbiased comparison, as not only patients with proven UTI, or without UTI, were chosen as control. Another weakness of our study is the lack of information on typical clinical symptoms or the presence of vaginal discharge in the medical record, which is important to assess clinical relevance of microbiological findings.

Despite these limitations, our study also has strengths. To our knowledge, this is the first study systematically addressing the possible role of *G. vaginalis* in urine cultures of renal transplant recipients. Although previous studies have described the presence of *G. vaginalis* in urine specimens of renal transplant recipients, assessment of clinical relevance has so far been limited to individual cases and small case series [13, 43–46]. Furthermore, the use of a clinically comparable transplant control group allowed comparison within a similar clinical setting.

## Conclusions

Taken together, our findings neither definitively confirm nor exclude *G. vaginalis* as a clinically relevant urinary pathogen in kidney transplant recipients. Detection of *G. vaginalis* should prompt careful clinical assessment integrating symptoms, inflammatory findings and competing microorganisms.

## Data Availability

The datasets generated during the current study are available from the corresponding author on reasonable request.
